# Transmural difference in myocardial damage assessed by layer-specific strain analysis in patients with ST elevation myocardial infarction

**DOI:** 10.1038/s41598-020-68043-w

**Published:** 2020-07-06

**Authors:** Sua Kim, Dong-Hyuk Cho, Mi-Na Kim, Soon-Jun Hong, Cheol Woong Yu, Do-Sun Lim, Wan Joo Shim, Seong-Mi Park

**Affiliations:** 1 Department of Critical Care Medicine, Korea University Ansan Hospital, Korea University College of Medicine, Ansan, Republic of Korea; 2 Division of Cardiology, Department of Internal Medicine, Korea University Anam Hospital, Korea University College of Medicine, 73, Inchon-ro, Seongbuk-gu, Seoul, 02841 Republic of Korea

**Keywords:** Medical research, Myocardial infarction

## Abstract

We performed layer-specific strain analysis with speckle-tracking echocardiography to investigate the transmural difference of myocardial damage as the predicting factor for the viability of damaged myocardium in patients with ST segment elevation myocardial infarction (STEMI). We analysed patients with acute STEMI who had undergone primary percutaneous coronary intervention and echocardiography within 24 h from the intervention and 2 months after the event. Segmental strains of the left ventricular (LV) endocardium, myocardium, epicardium, and strain gradient (SG) between the endocardium and epicardium were evaluated. In 34 patients, 112 akinetic/dyskinetic and 94 hypokinetic segments were observed among 612 segments of the LV at baseline, and 65 akinetic/dyskinetic segments had viability. In our study, layer-specific strains were gradually deteriorated by their wall motion. SG was augmented in the hypokinetic segments where inhomogeneous wall motion impairment was progressed. SG in the akinetic/dyskinetic segments was different between the viable and non-viable myocardium and was maintained in viable segments. We therefore believe that significantly reduced SG is indicative of irreversible transmural damage in the acute stage of STEMI and can be suitably used as a parameter for predicting myocardial viability.

## Introduction

After an acute myocardial infarction (AMI), a substantial part of the damaged myocardium recovers from the dysfunction^1–3^, and the subsequent development of heart failure and cardiac death after AMI depends on the extent of myocardial damage. Therefore, the viability of an akinetic or dyskinetic but stunned myocardium should be assessed for the long-term prognosis of patients with AMI^4,5^.

The left ventricular (LV) myocardium is composed of three layers, and there are transmural inhomogeneity in a damaged myocardium with AMI. When a coronary artery occlusion develops, myocardial damage starts from the endocardium and progresses as a wave front to the epicardium^6^. Within 60 min, the inner third of the LV wall becomes irreversibly injured; and within 6 h, the transmural extent of infarction is observed in the absence of collateral flow. Therefore, the difference in myocardial function between the endocardium and the epicardium could be associated with the presence of coronary artery disease^7^ and is expected to reflect irreversible myocardial damage. Recently, speckle-tracking echocardiography has been acknowledged to evaluate myocardial systolic function more accurately than the conventional echocardiography^8^. A detailed layer-specific analysis of LV function is possible with strain analysis, which enables separate analysis of endocardial myocardial, and epicardial deformation. This method allows transmural evaluation of myocardial damage in patients with AMI^9,10^.

We hypothesized that the difference in myocardial strain between the endocardium and epicardium (strain gradient, SG) of the damaged segment could be indicative of the transmural extent of myocardial damage. Parameters such as SG and layer-specific segmental strain could be important prognostic factors to predict the viability of the akinetic or dyskinetic segment. Therefore, in this study, we performed layer-specific strain analysis with speckle-tracking echocardiography to investigate the association of strain parameters and the viability of damaged myocardium in patients with AMI.

## Results

### Baseline characteristics of the patients and conventional echocardiography results

A total of 34 patients with ST segment elevation myocardial infarction (STEMI) were enrolled in the study. The mean age was 60 ± 11 years, and 28 patients were men. Twenty-five patients had anterior wall myocardial infarction. Peak CK-MB level was 229.1 ± 128.6 μg/L. Their ejection fraction was 50.2 ± 9.9% and global longitudinal strain of left ventricle was -10.9 ± 4.3% at baseline (Table 1). On follow-up transthoracic echocardiography, the ejection fraction (54.3 ± 10.0%, *p* = 0.02) and global longitudinal strain (− 15.0 ± 8.4%, *p* < 0.05) significantly improved.

**Table 1:** Baseline characteristics of patients and cardiac function evaluated with transthoracic echocardiography.

Age (years)	60 ± 11
Sex (M/F)	28/6
Hypertension	17
Diabetes mellitus	10
Smoking	19
Systolic blood pressure (mmHg)	118 ± 17
Diastolic blood pressure (mmHg)	75 ± 14
Heart rate (/min)	81 ± 12
Peak CK-MB (μg/L)	229.1 ± 128.6
Troponin-I (ng/mL)	2.72 ± 6.40
NTproBNP (pg/mL)	855.2 ± 2,817.5
hsCRP (mg/L)	4.4 ± 6.9
LVESV (mL)	39.6 ± 12.8
LVEDV (mL)	78.9 ± 17.7
EF (%)	50.2 ± 9.9
IVSd (mm)	11.0 ± 1.8
PWd (mm)	9.4 ± 2.0
E (cm/s)	58.2 ± 16.6
A (cm/s)	63.8 ± 16.2
DT (ms)	165.5 ± 54.9
E/e’	12.4 ± 4.6
GLS (%)	− 10.9 ± 4.3

*ESV* end systolic volume, *EDV* end diastolic volume, *EF* ejection fraction, *IVSd* diastolic interventricular septal thickness, *PWd* diastolic posterior wall thickness, *E* early diastolic mitral inflow, *A* end diastolic mitral inflow, *DT* descelerating time, *GLS* global longitudinal strain.

### Segmental layer-specific strain of normokinetic, hypokinetic and akinetic or dyskinetic segments in the baseline study

There were 405 normokinetic segments, 95 hypokinetic segments, and 112 akinetic or dyskinetic segments among the 612 segments from 34 patients. In normokinetic segments, longitudinal strain (LS) increased from the epicardial (LSepi) layer to the myocardial (LSmyo) and endocardial (LSepi) layers (LSendo, LSmyo, and LSepi were − 16.06 ± 6.54%, − 14.67 ± 5.30%, and − 13.60 ± 5.02%, respectively) and this trend was also observed in hypokinetic segments (− 8.92 ± 7.48%, − 6.87 ± 5.89%, and − 5.76 ± 4.97%, respectively) and akinetic or dyskinetic segments (− 1.35 ± 7.18%, − 0.56 ± 5.70%, and − 0.26 ± 5.02%, respectively).

**Figure 1:**
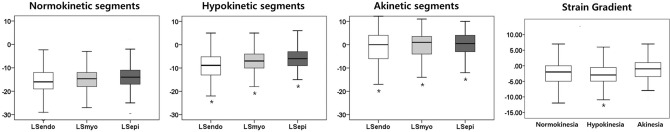
Layer-specific segmental strains and strain gradient between normokinetic, hypokinetic, and akinetic or dyskinetic segments in the baseline echocardiographic evaluation. *p < 0.05 compared to segmental strain in each layer and strain gradient of normokinetic segments.

Segmental strains in each layer gradually increased by their wall motion from akinetic or dyskinetic segments to hypokinetic and normokinetic segments (*p* < 0.001 for LSendo, LSmyo and LSepi). However, SG of the hypokinetic segment was significantly augmented in compare to SG of normokinetic segments (-2.96 ± 7.78% versus − 3.30 ± 4.24%, *p* = 0.017); there was no significant difference in SG between normokinetic segments and akinetic or dyskinetic segments (*p* = 1.00) (Table 2, Fig. 1).

**Table 2:** Layer-specific segmental strains in akinetic or dyskinetic, hypokinetic and normokinetic segments at baseline evaluation.

	Normokinetic segments (405)	Hypokinetic segments (95)	Akinetic/dyskinetic segments (112)	p-value*	p-value Normokinetic vs Hypokinetic	p-value Normokinetic vs Akinetic/dyskinetic
LSendo (%)	− 16.06 ± 6.54	− 8.92 ± 7.48	− 1.35 ± 7.18	< 0.001	< 0.001	< 0.001
LSmyo (%)	− 14.67 ± 5.30	− 6.87 ± 5.89	− 0.56 ± 5.70	< 0.001	< 0.001	< 0.001
LSepi (%)	− 13.60 ± 5.02	− 5.76 ± 4.97	− 0.26 ± 5.02	< 0.001	< 0.001	< 0.001
Strain gradient (%)	− 2.96 ± 7.78	− 3.30 ± 4.24	− 1.09 ± 3.50	0.015	0.017	1.00

*LSendo* segmental strain of endocardium, *LSmyo* segmental strain of myocardium, *LSepi* segmental strain of epicardium.

*Significance evaluation using linear mixed model for the influence of wall motion on strain parameters.

### Segmental layer-specific strains and segmental viability

**Figure 2:**
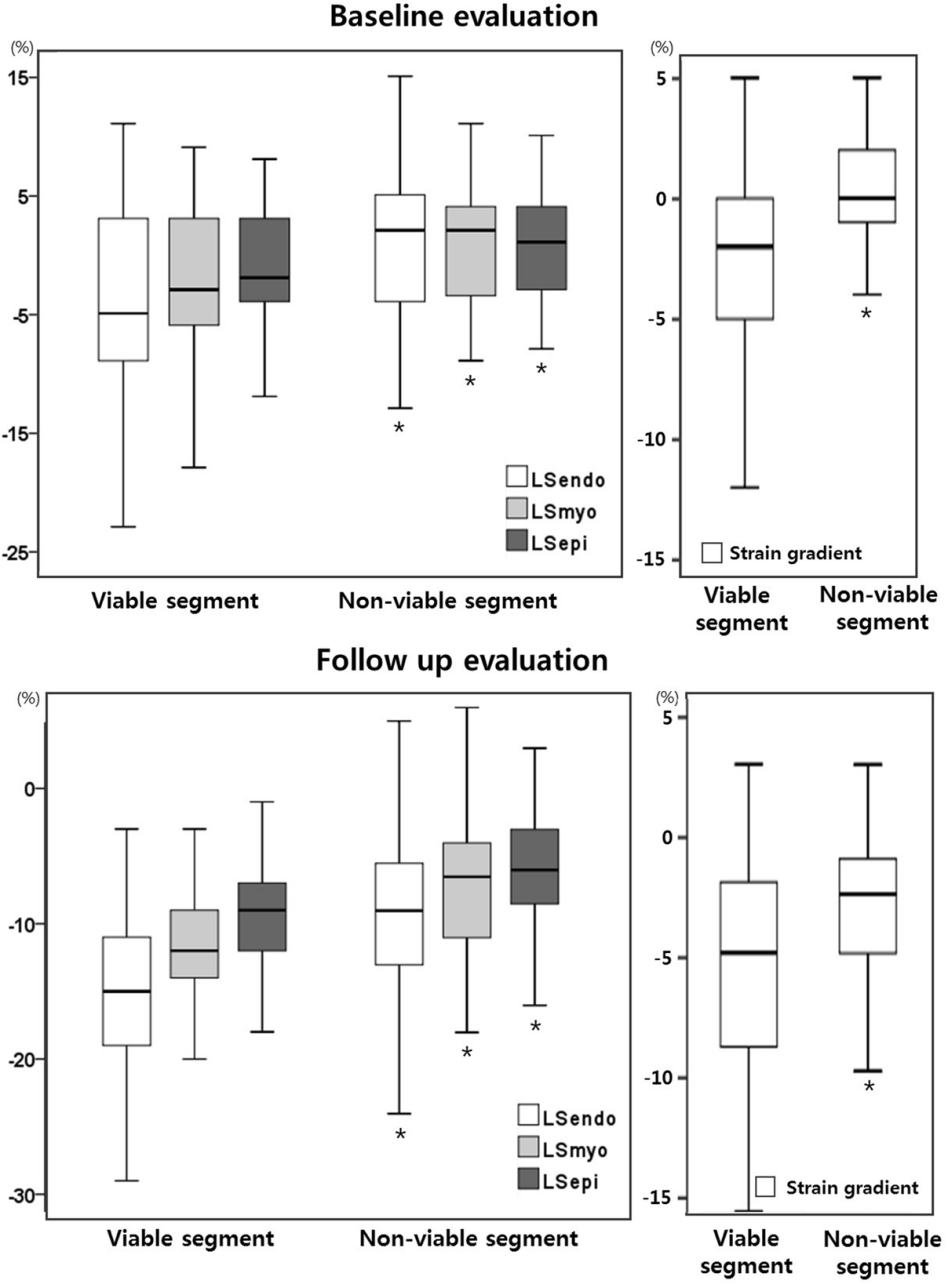
Layer-specific segmental strains and strain gradient of viable and non-viable segments among akinetic or dyskinetic segments in the baseline and follow up echocardiographic evaluation. *p < 0.05 compared to segmental strain of the same layer and strain gradient of viable segments.

When we evaluated the influence of viability on layer-specific segmental strains of akinetic or dyskinetic segments, segmental strain was significantly greater in the viable segment in all three layers (viable versus non-viable: LSendo, − 3.48 ± 7.39% versus 1.60 ± 5.77%, *p* = 0.001; LSmyo, − 1.92 ± 5.95% versus 1.32 ± 4.79, *p* = 0.011; LSepi, − 1.22 ± 5.23% versus 1.06 ± 4.43%, *p* = 0.043). SG was also significantly greater in viable segments (− 2.26 ± 3.63% versus 0.53 ± 2.56%, *p* < 0.001). In the follow-up study, segmental strains and SG of viable segments were significantly greater than those of non-viable segments (Table 3, Fig. 2). Subsequently, the influence of the viability on layer-specific segmental strains in the akinetic or dyskinetic segment was assumed from the linear mixed model. Estimate of the fixed effect of the viability on LSendo was 3.73 and that on SG was 2.49. (Table 4).

**Table 3:** Layer-specific segmental strains and strain gradient according to the viability in akinetic or dyskinetic segments at baseline and follow-up study.

	Baseline study			Follow-up study		
	Viable segments (65)	Non-viable segments (47)	p-value*	Viable segments (65)	Non-viable segments (47)	p-value*
LSendo (%)	− 3.48 ± 7.39	1.60 ± 5.77	0.001	− 15.25 ± 6.04	− 8.42 ± 6.00	< 0.001
LSmyo (%)	− 1.92 ± 5.95	1.32 ± 4.79	0.011	− 11.72 ± 4.53	− 6.67 ± 5.01	< 0.001
LSepi (%)	− 1.22 ± 5.23	1.06 ± 4.43	0.043	− 9.45 ± 4.07	− 5.46 ± 4.59	< 0.001
Strain gradient (%)	− 2.26 ± 3.63	0.53 ± 2.56	< 0.001	− 5.80 ± 4.40	− 2.96 ± 3.16	< 0.001

*LSendo* segmental strain of endocardium, *LSmyo* segmental strain of myocardium, *LSepi* segmental strain of epicardium.

*Significance evaluation using linear mixed model for the influence of viability on strain parameters.

**Table 4:** Fixed effect of segmental viability on layer-specific segmental strains and strain gradient using linear mixed model.

	Estimate	Standard error	t	95% confidence interval	p-value
LSendo	3.73	1.04	3.57	1.66–5.81	0.001
LSmyo	2.26	0.87	2.59	0.53–4.00	0.011
LSepi	1.66	0.81	2.05	0.05–3.26	0.043
Strain gradient	2.49	0.59	4.21	1.32–3.66	< 0.001

*LSendo* segmental strain of endocardium, *LSmyo* segmental strain of myocardium, *LSepi* segmental strain of epicardium.

**Figure 3:**
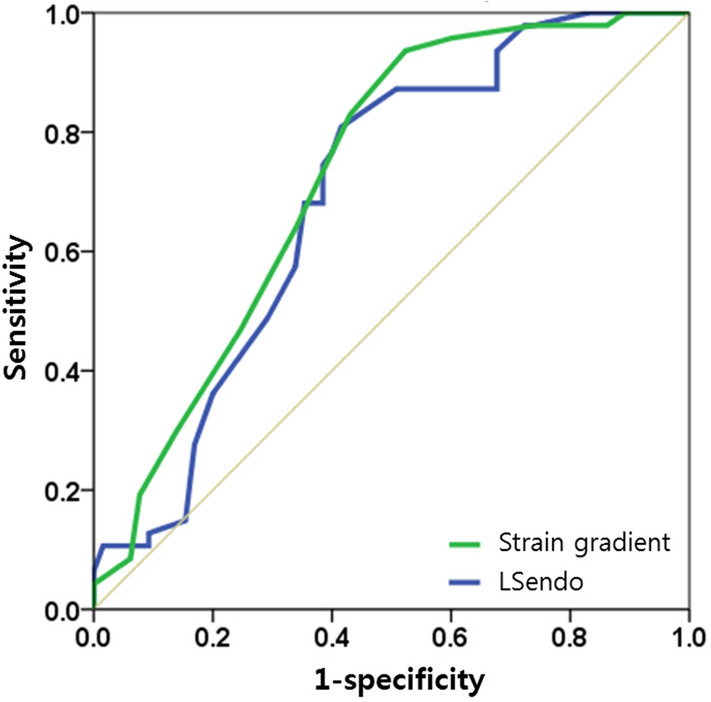
Receiver operating characteristic (ROC) curve for the segmental viability of akinetic or dyskinetic segments. *LSendo* segmental strain of endocardium. Area under the ROC curve is 0.69 for LSendo and 0.73 for strain gradient.

Finally, we performed receiver operating characteristic curve (ROC) analysis to evaluate the predictability of viability with layer-specific segmental strains (Fig. 3). Area under the ROC curve of SG was greater than that of LSendo (0.73 versus 0.69).

### Reproducibility of layer-specific segmental strain values

ICCs for intraobserver and interobserver variability of LSendo were 0.96 (0.948–0.971, p < 0.0001) and 0.910 (0.881–0.932, p < 0.0001), of LSmyo were 0.970 (0.960–0.978, p < 0.0001) and 0.932 (0.910–0.949, p < 0.0001), of LSepi were 0.968 (0.958–0.976, p < 0.001), and 0.940 (0.920–0.955, p < 0.0001), and of strain gradient were 0.834 (0.784–0.874, p < 0.0001) and 0.708 (0.672–0.774, p < 0.001). The intraobserver and interobserver variability was excellent for LSendo, LS myo and LSepi and good for strain gradient.

## Discussion

To the best of our knowledge, this is the first study on the assessment of LV function with layer-specific strain analysis in patients with STEMI undergoing primary percutaneous coronary intervention. The main findings of this study are (i) segmental strains of damaged LV gradually deteriorated with their wall motion, both in the endocardium and epicardium, and LSs were significantly different in each layer in normokinetic, hypokinetic and akinetic or dyskinetic segments; (ii) SG was augmented in hypokinetic segments where inhomogeneous wall motion impairment was progressed; (iii) akinetic or dyskinetic but viable segments exhibited higher LSs in all three layers and SG than non-viable segments exhibited; (iv) SG was only different in akinetic or dyskinetic segments and showed the best predictability for segmental viability.

Viability of damaged myocardium after myocardial infarction is an important prognostic factor^11^ because of its influence on LV function, symptoms of heart failure, and cardiovascular outcome when revascularization and medical therapy are properly performed^12–14^. Therefore, there have been several efforts to predict the viability of LV using non-invasive imaging modalities. Nuclear imaging^15^, ultrasonography^16,17^, and magnetic resonance imaging^18^ provided optimal values for predicting myocardial viability with good sensitivity and specificity. However, some of these modalities are very expensive, and require patients to be administered a radionuclide to enhance contrast for evaluation. Moreover, nuclear imaging and dobutamine stress echocardiography require stress to be given to the patient^19^, which may be inappropriate and harmful in acute stages of myocardial infarction^20^. Therefore, evaluation using such methods is not possible in patients with AMI. In this study, myocardial viability was evaluated using echocardiography without additional injection of contrast agents or stressors. Speckle-tracking analysis requires only a two-dimensional echocardiographic image of proper quality, which does not harm the patient, yet, provides additional information to the physician and patient^20,21^.

Myocardial injury due to ischemic heart disease starts from the endocardium, and spreads to the myocardium and epicardium due to the epicardial location of the coronary arteries and LV systolic pressure gradient during systole, which is called the wave front phenomenon^22^. Therefore, the evaluation of transmurality of myocardial damage and the degree of endocardial damage play an important role in the prognosis of coronary artery disease. Detection of myocardial damage limited to the endocardial layer without significant decrease in LV global function suggested the high probability of the presence of coronary artery disease in patients with chest pain^23^. In addition, the extension of myocardial damage to the epicardial layer suggested poor prognosis of ischemic heart disease leading to irreversible myocardial damage^21,24^. Likewise, an independent analysis of endocardial and epicardial strain and the evaluation of the difference between them are expected to discriminate the transmurality of myocardial damage in coronary artery disease. In this study, a gradual decrease of segmental strains was observed in the myocardium with deterioration of wall motion from normokinesia to hypokinesia and akinesia or dyskinesia. However, SG was rather increased in hypokinetic segment where wall motion was inhomogeneously impaired; it was not changed in the akinetic segments. This suggests that transmurality of the wall motion impairment only influenced the SG. SG was significantly decreased only in non-viable myocardium even in the akinetic or dyskinetic segments.

Evaluation of the global function using parameters such as ejection fraction or global longitudinal strain yield very small changes in these values because they incorporate both damaged and undamaged myocardium. Therefore, the evaluation of regional myocardial function has been reported to have superior prognostic implications in patients with acute myocardial infarction^10,25^. In our study, change in ejection fraction between the baseline and follow-up studies was approximately 4.08 ± 7.93%, change in GLS was 4.29 ± 8.47%, and the changes in segmental strain values of damaged myocardium were much greater. Therefore, we expect that risk assessment for viability with segmental strain parameters provides detailed information with no additional image acquisition or hazard to the patients in clinical practice.

The total number of patients in this study was small. However, the primary outcome of the study was segmental viability, which was evaluated in 112 akinetic or dyskinetic segments using linear mixed model. And, the statistical power of the analysis was enough to evaluate the influence of viability on segmental strain values and strain gradient especially in LSendo and SG; > 0.955 and 0.985 for each. Even in a patient with severely damaged left ventricle, the undamaged segments have normal segmental strain values whereas the damaged segments have worse result. Therefore, our evaluation may not be appropriate for the direct clinical prognosis prediction. Although we could not predict the risk of major adverse cardiac events with this evaluation, we thoroughly evaluated the viability of damaged segments after acute STEMI. We excluded patients who exhibited cardiac arrhythmia including atrial fibrillation for more accurate analysis of layer-specific strain and patients who did not have a follow-up image including those who died before the follow-up study. This implies that we may have excluded patients with more severe disease.

## Conclusion

Layer-specific segmental strain and SG were significantly different between normokinetic segments and akinetic or dyskinetic segments. Transmural difference represented by SG was increased in the hypokinetic segments where inhomogeneous wall motion impairment was progressed, but was maintained in the akinetic or dyskinetic segments. SG in the akinetic or dyskinetic segments was significantly reduced in non-viable segments and showed significant predictability for segmental viability. Therefore, SG in the acute stage of STEMI can be suitably used as a parameter for predicting myocardial viability.

## Methods

### Study population

Patients who visited the emergency department due to chest pain and diagnosed with STEMI were screened. If chest pain started within 24 h of the visit to the emergency room and primary percutaneous coronary intervention was performed, the patients were enrolled for the study. Patients with a history of coronary artery disease, structural heart disease, or cardiac arrhythmia, who did not undergo follow-up imaging, and did not give informed consent were excluded. This study was approved by Institutional Review Board of Korea University Anam Hospital, Seoul, South Korea and performed in accordance with relevant guidelines and regulations in accordance with the Declaration of Helsinki.

### Echocardiography

Both conventional two-dimensional echocardiography and speckle-tracking echocardiography were performed at baseline and follow-up. Baseline evaluation was performed within 24 h of revascularization, and follow-up echocardiography was performed 6–10 weeks after revascularization. All echocardiographic measurements were performed using a commercially available ultrasound system (VIVID-E9, GE Vingmed Ultrasound AS, Horten, Norway). Chamber diameter and wall thickness were measured directly from two-dimensional echocardiographic images, and LV mass was evaluated by linear measurements using the formula recommended by the European Association of Cardiovascular Imaging^26^. LV hypertrophy was defined as LV mass index of > 95 g/m^2^ in women and > 115 g/m^2^ in men. LV ejection fraction was calculated using the modified Simpson’s method from apical four- and two-chamber views, and the LV volume was measured at the ventricular end systole and end diastole. Mitral inflow was obtained using pulse-wave Doppler echocardiography with the sample volume between mitral leaflet tips during diastole. Myocardial movement was categorized into three grades: normokinesia, hypokinesia and akinesia or dyskinesia. Viability was analysed in terms of the improvement in segmental movement of akinetic or dyskinetic segment by more than a grade during the follow-up study; for example, akinesia or dyskinesia to hypokinesia or normokinesia.

### Layer-specific segmental strain analysis

To evaluate the layer-specific strain, apical four-chamber, two-chamber, and long-axis views were recorded with a frame rate of 60–80 frame/s during baseline and follow-up echocardiographic evaluations. Then, strain analysis was performed with the off-line software (EchoPAC PC, GE Medical Systems). After manual tracing of the endocardial border of the LV in three apical images, endocardial, myocardial, and epicardial segmental and global LSs were obtained using LSendo, LSmyo, and LSepi, respectively, for segmental layer-specific LS. Segmental SG between the endocardium and epicardium was also calculated according to the difference in LSendo and LSepi in each segment. For the analysis of segmental viability, we checked the correlation between the culprit coronary artery and the region of the akinetic or dyskinetic segment.

### Statistical analysis

Continuous variables were expressed as mean ± standard deviation, and categorical variables as frequencies and percentages. Segmental strain parameters between layers and between baseline and follow-up studies were compared using repeated measure ANOVA. The influence of wall motion and viability on segmental strain parameters were evaluated using linear mixed model. We performed receiver operating characteristic curve analysis to evaluate the predictability of the viability using layer-specific segmental strains and SG. To assess the reproducibility of the variables, LSendo, LSmyo, LSepi and strain gradient were repeatedly evaluated on the same echocardiographic images of ten patients. Then, we assessed the intra-observer variability and inter-observer variability using intraclass correlation coefficient (ICC). All statistical analysis was performed with SPSS version 20.0 (IBM, Armonk, New York, USA) and SAS version 9.4 (SAS institute, Cary, NC, USA).

## Data Availability

The datasets generated during and/or analyzed during the current study are available from the corresponding author on reasonable request.
